# Unravelling the Potential of Fungal Division of Labour in the Laccase Producer *Coriolopsis trogii* MUT3379 Through Protoplast Formation and Regeneration

**DOI:** 10.3390/jof11120890

**Published:** 2025-12-17

**Authors:** Luca Mellere, Adriana Bava, Jean Armengaud, Francesca Berini, Flavia Marinelli, Giovanna Cristina Varese, Federica Spina, Fabrizio Beltrametti

**Affiliations:** 1 Department of Biotechnology and Life Sciences, University of Insubria, 21100 Varese, Italy; lmellere@uninsubria.it (L.M.); f.berini@uninsubria.it (F.B.); flavia.marinelli@uninsubria.it (F.M.); 2 BioC-CheM Solutions S.r.l., Insubrias BioPark, 21040 Gerenzano, Italy; abava@bioc-chemsolutions.com; 3 Département Médicaments et Technologies pour la Santé (DMTS), CEA, INRAE, SPI, Université Paris-Saclay, 30200 Bagnols-sur-Cèze, France; jean.armengaud@cea.fr; 4 Mycotheca Universitatis Taurinensis (MUT), Department of Life Sciences and Systems Biology, University of Turin, Viale Mattioli, 25, 10125 Turin, Italy; cristina.varese@unito.it (G.C.V.); federica.spina@unito.it (F.S.)

**Keywords:** laccase, oxidase, fungi, division of labour, fermentation, protoplasts

## Abstract

The white-rot fungus *Coriolopsis trogii* MUT3379 produces Lac3379-1 laccase at high yields due to the previous development of a robust fermentation process. Throughout the extended use of this strain, we observed the occurrence of substrate-specific guaiacol and ABTS (2,2’-azino-bis(3-ethylbenzothiazoline-6-sulphonic acid)) oxidizing enzymes other than Lac3379-1 Since we did not succeed in producing these enzymes in significant amounts by conventional strain selection and fermentation tools, we developed an approach based on protoplast preparation and regeneration to isolate stable producers of these alternative oxidative enzymes from the complex multinucleate mycelium of *C. trogii* MUT3379. A cost-effective and efficient protocol for protoplast preparation was developed by using the enzymatic cocktail VinoTaste Pro by Novozymes. A total of 100 protoplast-derived clones were selected and screened to produce laccases and other oxidative enzymes. A variable spectrum of oxidative activity levels, including both high and low producers, was revealed. Notably, a subset of clones exhibited diverse guaiacol/ABTS positive enzymatic patterns. These findings suggest that it is possible to isolate different lineages from the mycelium of *C. trogii* MUT337, each producing a distinct pattern of oxidative enzymes. This highlights the potential of protoplast-mediated genome separation to uncover novel metabolic traits that would otherwise remain cryptic. These data hold outstanding significance for accessing and producing novel oxidative enzymes from native fungal populations.

## 1. Introduction

Fungi of the phylum Basidiomycota are widely employed as cell factories to produce a variety of biomolecules ranging from antibiotics to enzymes [1]. In nature, Basidiomycota grow forming complex structures alternating vegetative and reproductive phases and involving the formation of vegetative mycelium (or hyphae) and complex fruiting bodies dedicated to spore production [2,3]. Vegetative hyphae are ultimately destined to die and have been previously described as a “sterile caste” [4,5,6,7], offering the advantage of limiting the metabolic cost of complex biosynthesis pathways to only a certain fraction of the mycelium [6]. This organization is considered an example of division of labour, in which phenotypically distinct cell populations [8] serve specific functions and synergistically interact with each other to sustain growth and species preservation, especially when resources become limited [8,9].

Division of labour in filamentous microorganisms may implicate metabolic differentiation. For instance, the sporulating hyphae of *Aspergillus niger* do not secrete proteins, suggesting that this process is confined to vegetative cell lineages within the mycelium. Consistently, deletion of the sporulation-related gene *flbA* in *A. niger* results in a more homogeneous secretion of proteins throughout the whole mycelium, meanwhile increasing the complexity of the fungal secretome compared to the wild-type strain [10]. Differential enzyme secretion within the mycelium was also previously reported in the model basidiomycete *Phanerochaete chrysosporium*, both in solid cultures [11] and in submerged fermentations [12]. Other phenomena might contribute to the metabolic variability in filamentous fungi. For instance, different lineages may generate, through hyphal fusion, viable heterokaryons in which genetically different nuclei coexist [13]. This may potentially result in functional diploidy, but it could also increase the genetic diversity through mitotic recombination [14]. In addition, spontaneous mutations frequently occur in filamentous fungi due to their intrinsic genomic plasticity [15]. While metabolic variability and genome plasticity [16] are essential traits for the survival of the fungal species in natural environments, they might result in strain degeneration and poor process reproducibility in laboratory and industrial settings, limiting the biotechnological use of filamentous fungi as cell factories [17,18].

*Coriolopsis trogii* MUT3379 is a white-rot fungus producing and secreting one laccase that we previously named Lac3379-1 [19]. It was recently produced at high yield, developing a robust fermentation process in liquid cultures [19]. While in standard fermentation conditions, Lac3379-1 appears to be the only laccase produced by *C. trogii*, during our long-lasting use of this strain at industrial scale, we observed in zymograms the appearance of different bands showing guaiacol/ABTS oxidazing activity, in addition to the major spot attributable to Lac3379-1. Fungal laccases and other non-laccase oxidative enzymes are known to occur in multigene families and are often produced as isoenzymes, which may possess different enzymatic properties and/or activities [20]. We hence hypothesized that phenotypically distinct subpopulations in *C. trogii* mycelium have specialized to produce these alternative enzymatic patterns that, in standard fermentation conditions, are hindered by laccase Lac3379-1 high-level production. Besides the clear ecological meaning, the possibility to identify and efficiently produce alternative laccase or oxidative enzyme isoforms holds considerable industrial potential. Different isoforms can exhibit structural variations which may result in diverse enzymatic properties, such as different substrate specificity, redox potential, catalytic efficiency and stability [20]. Therefore, in this study, we investigated whether selecting subpopulations within the vegetative mycelium of *C. trogii* could help in exploring different stable oxidative enzyme profiles. From the technical point of view, filamentous fungi are mycelial and polynucleated microorganisms, and the simple replication of colonies, or plating them by dilution, does not easily allow phenotypically different cell separations. We therefore performed the mycelium enzymatic digestion to isolate protoplasts carrying a limited (possibly only one) number of nuclei, with the aim of separating the different genotypes present within the starting polynucleated mycelium. By promoting protoplast regeneration in appropriate conditions, it was possible to isolate variants of *C. trogii*, giving rise to phenotypically diverse clonal populations which were then studied with the final scope to improve industrial production of novel oxidative enzymatic profiles.

## 2. Materials and Methods

### 2.1. Cultivation of C. trogii MUT3379 and Production of Oxidative Enzymes

The fungal strain used in this project was *Coriolopsis trogii* MUT3379 from the collection Mycotheca Universitatis Taurinensis. The strain was isolated from rotting wood collected at “La Mandria” park in Turin (Italy). The fungal mycelium was preserved in a solution of nutrient glycerol, obtained by solubilizing 8 g/L of nutrient broth (DIFCO, Franklin Lakes, NJ, USA) and 200 g/L of glycerol (CARLO ERBA Reagents, Cornaredo, Italy) in demineralized water, and stored as Working Cell Banks (WCB) at −80 °C. The strain was routinely propagated by growing it on Malt Extract Agar (MEA; 20 g/L malt extract (Costantino & C, Favria, Italy), 20 g/L casein peptone (Organotechnie, La Courneuve, France), 20 g/L agar (HiMedia, Modautal, Germany)), at 25 °C for 7 days. Fermentation media used in this work were selected from the BCSMedDat database owned by BioC-CheM Solutions (https://www.bioc-chemsolutions.com), unless otherwise stated. They were prepared by solubilizing the requested raw materials in demineralized water while the pH was corrected by adding HCl or NaOH solutions, followed by sterilization at 121 °C for 20 min. All media components and reagents used were from Costantino & C. (Favria, Italy), CARLO ERBA Reagents (Cornaredo, Italy), Roquette (Lestrem, France), or Merck KGaA (Darmstadt, Germany), unless otherwise indicated.

Liquid cultures of *C. trogii* MUT3379 and the further derived clones were prepared by inoculating a mycelium plug of 1 cm^2^ grown on MEA plate, into a 500 mL baffled flask containing 100 mL of medium BCS218, supplemented with 75 μM CuSO_4_ to promote laccase production [19]. Fermentations were carried out at 25 °C and 150 rpm for 240 h, and 2 mL samples were collected at different time intervals and centrifuged at 16,000× *g* for 10 min at 25 °C. Solid State Fermentations (SSFs) were carried out in 75 cm^2^ Roux bottles containing 10 g of solid substrate (sawdust of different plant species including larch, grapevine, oak, chestnut, cherry tree, olive tree, apple tree, ash, laurel, fir, and ailanthus) inoculated by 50 mL of a liquid culture grown in BCS218 medium for 7 days at 25 °C and 150 rpm. After 21 days of incubation at 25 °C, the SSFs were amended with 100 mM Tris-HCl pH 8 and vacuum filtered.

Production of laccase and other ABTS oxidase enzymes was monitored in the culture supernatants from liquid cultures, or in the culture filtrates from SSF, by the activity assay on 2,2’-azino-bis(3-ethylbenzothiazoline-6-sulphonic acid) (ABTS) as substrate and by polyacrylamide gel electrophoresis (PAGE) in native and denaturing conditions, as described below. Fungal biomass growth in liquid cultures was measured by dry weight (g/L) of the pellet from 50 mL of culture after lyophilization.

### 2.2. Protoplast Preparation and Manipulation

Dispersed mycelium of *C. trogii* MUT3379 was obtained by culturing the strain in stationary liquid cultures of yeast-malt extract glucose medium (YMG; 4 g/L yeast extract, 10 g/L malt extract, 4 g/L glucose) at 25 °C for 7 days. The obtained biomass was centrifuged at 3000× *g* for 20 min, and the pellet was resuspended in the lysis solution based on VinoTaste Pro (at 25 or 50 g/L; Novozymes, Bagsværd, Denmark). Incubation was run up to 8 h at 37 °C, and protoplast formation was monitored by microscopic observations at regular intervals of 2 h. Protoplasts were then detached from residual mycelium clumps by thoroughly pipetting up and down, and they were then separated from residual hyphal fragments by filtration through glass wool. The protoplast solution was then centrifuged, and the protoplasts were resuspended in an appropriate hypertonic medium (BCS376; https://www.bioc-chemsolutions.com). The total protoplast number was determined by using a Petroff–Hausser counting chamber.

Mycelium regeneration from protoplasts was performed using the overlay technique previously applied to actinomycetes, proposed by Shirahama et al. [21]. The protoplast suspensions were seeded on a hypertonic modification of YMG medium (YMG-m1; 4 g/L yeast extract, 10 g/L malt extract, 4 g/L glucose, 80 g/L sucrose, 20 mM calcium chloride, 10 mM magnesium chloride, 18 g/L agar (BD Difco, Franklin Lakes, NJ, USA)) and then overlaid with low melting agar YMG (YMG-m2; 4 g/L yeast extract, 10 g/L malt extract, 4 g/L glucose, 20 mM calcium chloride, 10 mM magnesium chloride, 4 g/L agar low-melting (Merck KGaA, Darmstadt, Germany)). To evaluate the presence of residual hyphae in the protoplast suspensions, control plates (with YMG as under layer and YMG-m2 as the upper layer) were seeded. In these solid media, devoid of high concentrations of sucrose, only hyphal cells, but not protoplasts, were able to grow. Finally, a pool of 100 colonies regenerated from protoplasts was replicated on MEA plates, and each clone was then tested for ABTS oxidizing activity (ABTS assay) and for the presence of different guaiacol-oxidizing enzymes in non-denaturing conditions (NATIVE PAGE), following the cultivation protocol described above.

### 2.3. Enzyme Assays

ABTS oxidizing enzyme activity was routinely assayed spectrophotometrically at 25 °C (unless otherwise stated), using a V530 Jasco spectrophotometer (Easton, MD, USA) following the oxidation of ABTS (ε420 nm = 36 mM^−1^ cm^−1^) at 420 nm for 5 min. 0.5 mM ABTS was added to 50 mM sodium acetate, pH 4. One unit of activity was defined as the amount of enzyme that oxidizes 1 μmol of ABTS per min at 25 °C and is indicated as ABTS-U L^−1^ of culture broths from fermentation. In some experiments, ABTS oxidation was performed at 40, 50, 60, 70, 80, and 90 °C.

The oxidizing enzymatic activity of selected protoplast-derived clones was also assayed spectrophotometrically on guaiacol (ε468 nm = 12 mM^−1^ cm^−1^; guaiacol-U L^−1^) and 2,6-dimethylphenol (2,6-DMP, ε468 nm = 49.6 mM^−1^ cm^−1^; 2,6-DMP-U L^−1^). The oxidizing activities were monitored for 5 min at 25 °C in 50 mM sodium acetate buffer at pH 3, 4, 5, and 6, and in 50 mM HEPES at pH 6, 7, and 8.

### 2.4. NATIVE-PAGE and Zymograms

Polyacrylamide gel electrophoresis (PAGE) was performed under non-denaturing conditions (NATIVE-PAGE) to visualize laccase enzymes and/or isoforms and/or other oxidative enzymes secreted by the fungus in culture supernatants. Samples for analysis were prepared by centrifuging the grown fungal culture at 18,000 rcf for 10 min, followed by filtration on 0.22 μm membranes. Supernatant samples (5 to 20 μL) were then mixed with loading buffer and loaded on the gel. The analysis was carried out on 3% (*w*/*v*) acrylamide stacking gel, pH 6.8, and 14% (*w*/*v*) acrylamide gel for the running gel at pH 8.9. Tris-Glycine buffer 0.5×, pH 6.8, was used as the running buffer. For zymogram analysis, the enzyme visualization was performed with a solution of 2 mM guaiacol in 50 mM sodium acetate buffer, pH 5.

### 2.5. Tandem Mass Spectrometry

*C. trogii* secretomes were run on sodium dodecyl sulphate (SDS)-PAGE gels and visualized with Coomassie brilliant blue in standard conditions. The guaiacol-oxidizing protein band was excised as a single polyacrylamide band by using a scalpel and processed as already reported in Mellere et al. [19]. The protein was digested with trypsin, the resulting peptides were purified and analyzed by tandem mass spectrometry coupled to high-performance liquid chromatography (nanoLC-MS/MS) with a high-resolution Q-Exactive HF instrument (Thermo Scientific™, Waltham, MA, USA) coupled with an UltiMate 3000 LC system (Dionex-LC) essentially as previously described [22]. Briefly, the instrument was operated in data-dependent mode. Peptides were desalted on an Acclaim PepMap100 C18 precolumn (5 μm, 100 Å, 300 μm id × 5 mm), and then resolved on a nanoscale Acclaim PepMap 100 C18 column (3 μm, 100 Å, 75 μm id × 50 cm) with a 90 min gradient at a flow rate of 0.2 μL/min. The gradient was developed from 5% to 25% of (CH_3_CN, 0.1% HCOOH) over 75 min, and then from 25% to 40% over 15 min. Peptides were analyzed during scan cycles initiated by a full scan of peptide ions acquired from *m*/*z* 350 to 1500 at a resolution of 60,000 in the ultra-high-field Orbitrap analyzer, followed by high-energy collisional dissociation and MS/MS scans on the 20 most abundant precursor ions (Top20 method). For MS/MS fragmentation, only precursor ions with charge states of 2+ and 3+ were selected, using an AGC target of at least 1 × 10^5^ and a dynamic exclusion window of 10 s to enhance the detection of novel low-abundant analytes. MS/MS resolution was 15,000. For the interpretation, the assembled genome from *Coriolopsis trogii* C001 (GCA_020543525.1) was employed. Initially, all the possible stop-to-stop ORFs were predicted and transformed into polypeptide sequences with the same strategy as previously described [23]. This proteogenomic database was used to interpret the MS/MS spectra using The Mascot Daemon 2.6.1 search engine (Matrix Science, London, UK) with the following parameters: tolerance of 5 ppm for the parent ions and 0.02 Da for the fragmented ions, Carbamidomethyl (C) as fixed modification, deamidated (N, Q) and oxidation (M) as variable modification, and a maximum of 2 trypsin miss-cleavages. Peptides and proteins were identified with an FDR of 0.01 calculated from the relevant decoy database search. A DIAMOND similarity search was then performed to detect the most similar sequence present in the NCBI non-redundant database. Based on the preliminary results, the *C. trogii* C001 genome sequence, where the laccase compatible peptides mapped, was retrieved and analyzed with AUGUSTUS (http://augustus.gobics.de/, accessed on 9 December 2025) for the identification and assembly of exons into the putative full-length polypeptide. The putative protein was then aligned with ClustalW (http://www.clustal.org/clustal2/, accessed on 9 December 2025) to the LC-MS/MS identified peptides to identify the most probable deduced sequences. The identified sequences were then matched against the NCBI non-redundant protein database by use of BLASTP (https://blast.ncbi.nlm.nih.gov/) (accessed on 9 December 2025). For the attribution of CMC oxidases to CAZy subfamilies, a matching of the data from BLASTP comparison with the data of the Carbohydrates Active Website (www.cazy.org) was performed (accessed on 9 December 2025).

## 3. Results

### 3.1. Production of Laccases and Other Oxidative Enzymes in C. trogii MUT3379

**Figure 1:**
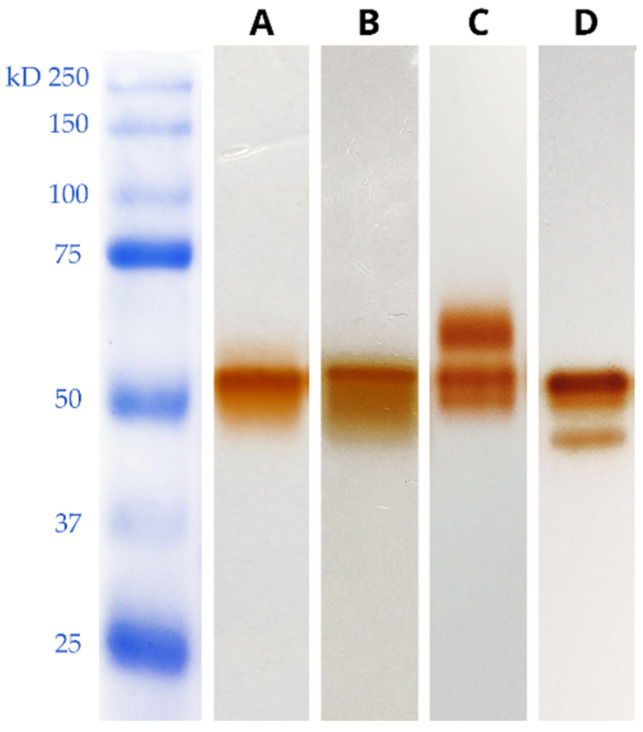
NATIVE-PAGE and zymogram analyses of the Lac3379-1 (**A**) and culture filtrates from SSFs on ailanthus (**B**), chestnut (**C**), and grapevine (**D**) sawdust. Oxidative enzyme bands were detected by incubation with 2 mM guaiacol.

Cultivating *C. trogii* MUT3379 in the liquid medium BCS218 led to the production into the broth of Lac3379-1 [19] as demonstrated by the single band in NATIVE-PAGE and in the corresponding zymogram using guaiacol as substrate (Figure 1A). Addition of different inducers, such as metal ions, aromatic and phenolic compounds, did not trigger the production of different laccase isoforms in *C. trogii* MUT3379, as indeed was reported in other filamentous fungi [24,25]. When *C. trogii* MUT3379 was grown in SSFs using sawdust from different tree species, in most of the cultivation conditions, a single defined band appeared on the gel after 21 days of incubation (Figure 1B), which was indistinguishable from Lac3379-1. However, in SSFs on grapevine and chestnut sawdust, a different enzymatic pattern, with different guaiacol positive bands other than Lac3379-1 was detectable (Figure 1C,D). Production of these alternative SSF-induced oxidases in quantitative amounts was unsuccessful, likely due to the predominance of Lac3379-1 activity in most of the fermentative conditions and in the presence of the commonly used inducers.

### 3.2. Protoplast-Derived Clone Isolation

**Figure 2:**
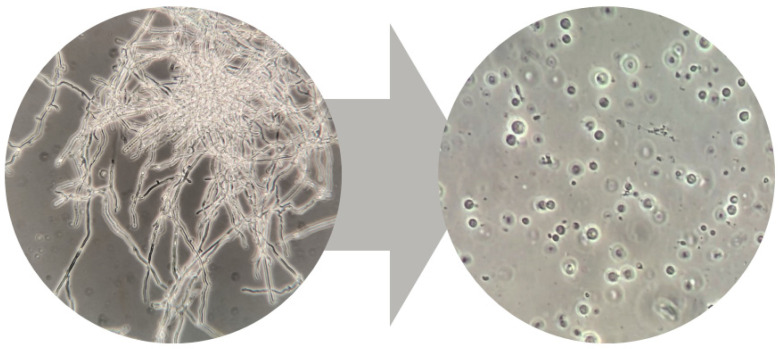
From hyphae to protoplasts in *C. trogii* MUT3379. Pictures were taken with an optical microscope (ZEISS, Oberkochen, Germany) at 40×, for mycelium (**left**) and 100× magnification for protoplasts (**right**).

Following the hypothesis that specific cell lineages within the complex *C. trogii* MUT3379 mycelium might produce a diverse pattern of laccases or other oxidative enzymes, we applied protoplast preparation and regeneration to possibly highlight these hindered phenotypes. To achieve efficient and homogeneous protoplast production, the enzymatic hydrolysis of the cell wall was performed by using the VinoTaste Pro enzymatic cocktail by Novozymes. The best results were obtained using 25 g/L of powder in hypertonic medium BCS376 after 6 h of incubation at 37 °C. In this condition, a total of 10^6^–10^7^ protoplasts/mL were obtained (Figure 2).

After 10 days of incubation, the protoplast percentage that reverted to filamentous state on the permissive medium YMG-m1 was estimated to be 5.8 ± 0.3%. No residual hyphal contamination was observed as no colonies grew on control YMG agar plates (non-permissive medium). A total of 100 protoplast-deriving colonies were selected, replicated on MEA agar plates and incubated for 7 days at 25 °C. The isolated lineages exhibited morphological differences when cultivated either in solid media (Supplementary Figure S1B–D) or in liquid cultures (Figure S2B–F). Cultivation of the control strain *C. trogii* MUT3379 and of the protoplast-deriving clones in liquid medium BCS218 added with 75 μM CuSO_4_, gave rise to a different culture broth pigmentation (from pale-yellow to orange and dark brown variants) associated with a variable mycelium morphology (from highly heterogeneous pellets in terms of both shape and dimension to more dispersed hyphae—Figure S2E—or homogeneous and well-defined round pellets—Figure S2F). Overall, these morphological analyses, although purely qualitative, confirm that protoplast preparation and regeneration can represent an efficient tool to separate different phenotypes from *C. trogii* mycelium.

### 3.3. Quantification of the Oxidative Activity in the Protoplast-Derived Clones

**Figure 3:**
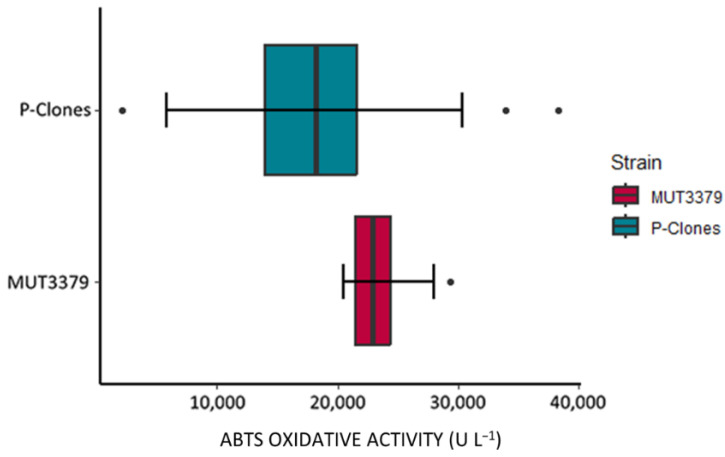
Boxplot of ABTS oxidizing activity (ABTS-U L^−1^) of the 100 protoplast-derived clones (P-Clones) and of a set of 16 fermentations of the parental strain MUT3379.

**Figure 4:**
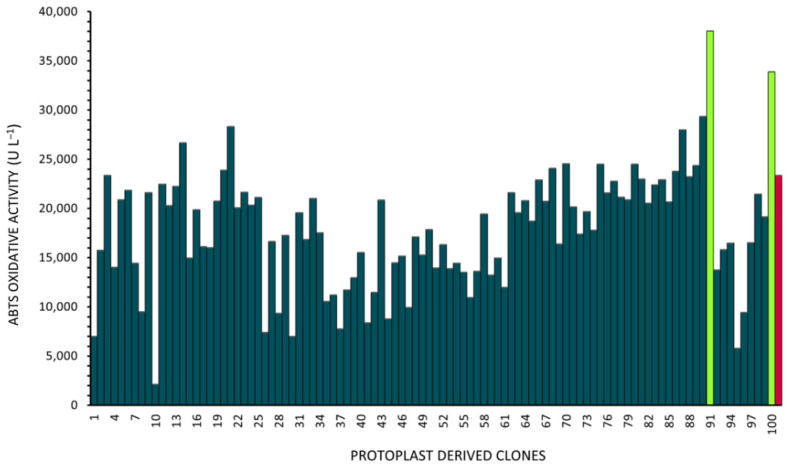
ABTS oxidizing activity (ABTS-U L^−1^) of the 100 isolated clones in medium BCS218–CuSO_4_ 75 μM. Highest producing clones evidenced in green, and control strain (average) in red.

After 240 h of cultivation in BCS218—75 μM CuSO_4_, the oxidizing activity on ABTS in the culture supernatants from the 100 protoplast-deriving clones was quantified. A variable range of activity levels was observed when compared with a set of standard fermentations deriving from the parental strain (Figure 3). Indeed, the average value of oxidizing activity achieved in the protoplast-derived population reached 18,000 ± 6038 ABTS-U L^−1^ while the set of standard fermentations gave 23,000 ± 2478 ABTS-U L^−1^. In comparison to the original strain, the distribution of the activity in protoplast-derived clones shifted towards the extremes, showing an increase in both low and high producers (out layers) (Figure 3 and Figure 4). Clone 91p and 100p showed the highest ABTS oxidizing activity overall, ranging from 33,000 to 38,000 ABTS-U L^−1^. On the other hand, clones 10p and 95p were found to be the lowest producers, highlighting the presence of lineages with scarce production/secretion capacities or with oxidizing activities undetectable by the standard assay in use (Figure 4).

**Figure 5:**
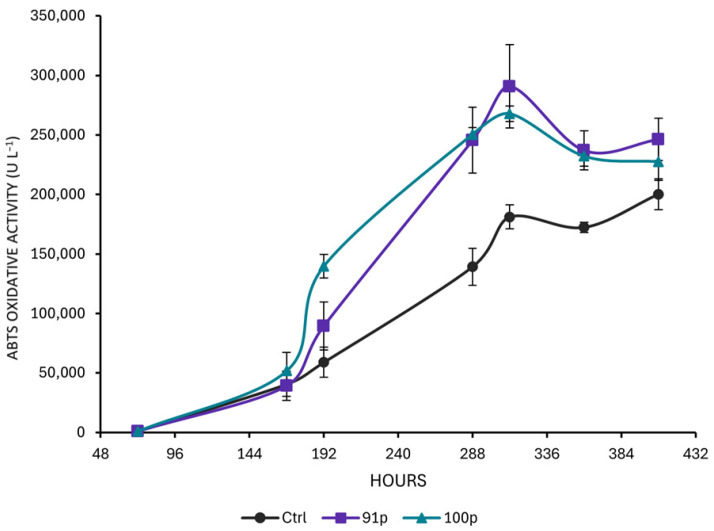
Production kinetics of parental strain MUT3379 (Ctrl) and protoplast-derived clones 91p and 100p, using the fermentation flow-sheet described in [19]. Results are the average ± standard deviations of three independent fermentations.

Oxidizing activity by the highest producing clones (91p and 100p) was then compared with the parental strain MUT3379 following the previously optimized fermentation flow-sheet [19]. Protoplast-derived clones 91p and 100p showed an improvement with respect to the parental strain, with an increase in activity of 45 ± 18% and 34 ± 3%, respectively (Figure 5). It is worth noting that the production kinetics in clones 91p and 100p was faster than in the control strain MUT3379 reaching the maximum of activity of ca. 291,000 ± 35,139 ABTS-U L^−1^ (for clone 91p) and 267,000 ± 6349 (for 100p) in 300 h vs. 200,000 ± 4218 ABTS-U L^−1^ in 408 h for the parental strain (Figure 5). The increase in ABTS-oxidizing activity in clones 91p and 100p was also confirmed in terms of ABTS-U g^−1^ of biomass. In this case, the best producer overall resulted in clone 100p with an average yield of 33,780 ± 3823 ABTS-U g^−1^ followed by clone 91p with a yield of 28,215 ± 960 ABTS-U g^−1^, both higher than the parental strain MUT3379 (24,305 ± 6239 ABTS-U g^−1^).

### 3.4. Comparison of the Oxidative Potential of the Protoplast-Derived Clones

**Figure 6:**
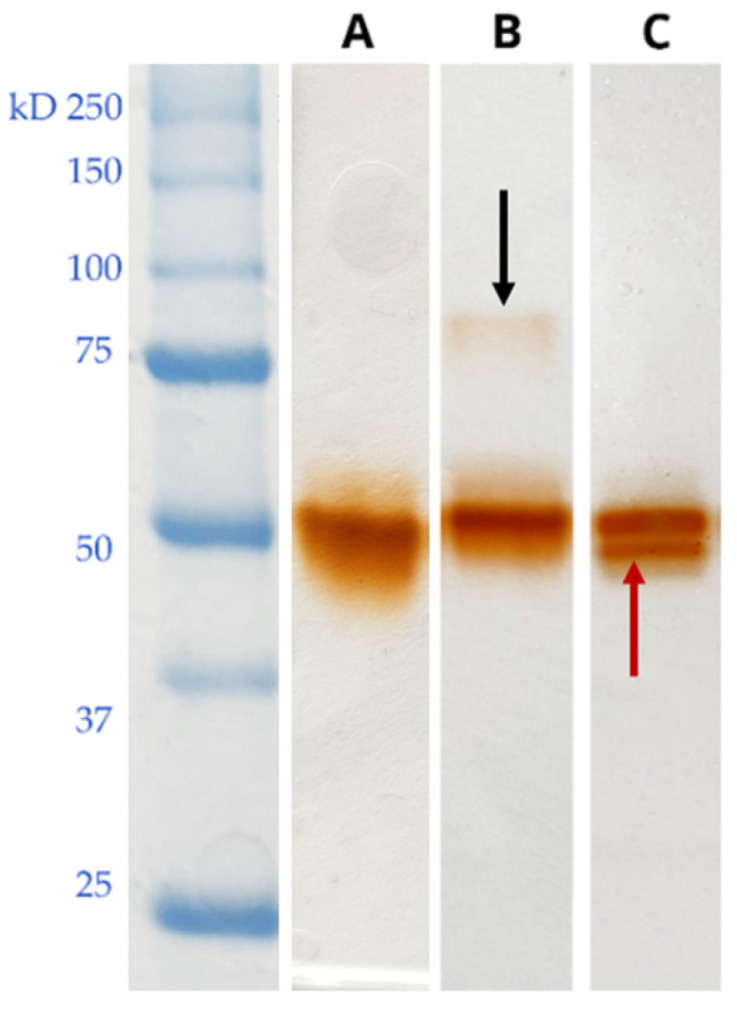
NATIVE-PAGE and zymogram analyses of culture supernatants from the parental strain MUT3379 (**A**), clone 6p (**B**), and clone 26p (**C**). Oxidative enzyme bands were detected by incubation with 2 mM guaiacol. Relevant differences from the parental zymograms are indicated by arrows.

Despite the variable activity levels, most of the protoplast-derived clones displayed the apparent production and secretion of a single predominant guaiacol-oxidizing enzyme (evidenced by NATIVE-PAGE analysis of culture supernatants developed with 2 mM guaiacol) reasonably identical to Lac3379-1 (Figure 6A). In a few protoplast-derived clones, zymograms instead revealed the presence of different guaiacol-positive bands. In culture supernatants of clones 26p, 28p, and 30p (exemplified by Figure 6C), a second band with a slightly lower molecular weight than that of Lac3379-1 was detectable (below indicated as putative oxidase low molecular weight or Ox-L). On the contrary, protoplast-derived clones 6p, 13p, and 14p displayed the presence of an additional guaiacol-oxidizing protein band with a higher molecular weight (exemplified by Figure 6B) (below indicated as putative oxidase high molecular weight or Ox-H).

**Figure 7:**
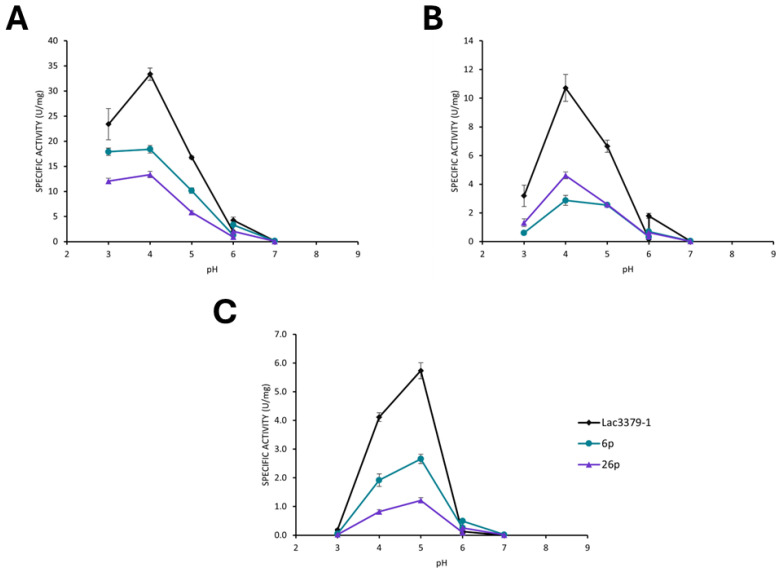
Enzymatic activities of Lac3379-1 in comparison to clones 6p and 26p supernatants on ABTS (**A**), 2,6-DMP (**B**), and guaiacol (**C**) at different pHs.

The secretomes of these clones were further characterized for their oxidative properties on ABTS, guaiacol, and 2,6-DMP and compared with the parental strain MUT3379. As shown in Figure 7, the protoplast-derived clones showed variable oxidative capacities on the different tested substrates, with an undefined trend that was not directly imputable to the presence of the different patterns observed in NATIVE-PAGE.

**Figure 8:**
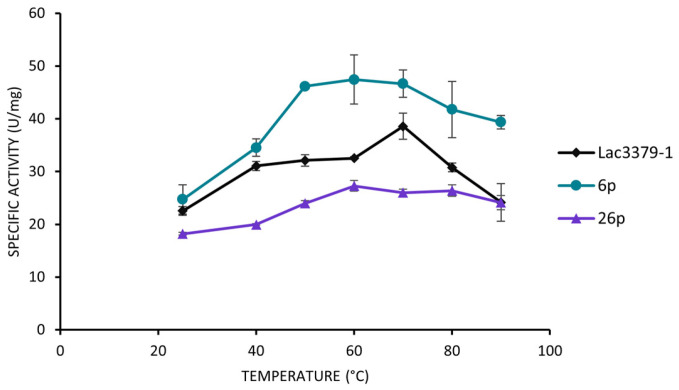
Enzymatic activity of Lac3379-1 compared to those of clones 6p and 26p supernatants on ABTS at different temperatures.

The oxidizing activities of the selected clones on ABTS were also compared at different temperatures. As reported in Figure 8, both clones 6p and 26p, exhibited variations in the specific activity depending on the experimental temperature. Clone 6p demonstrated a marked increase in activity as the temperature rose, suggesting a possible enhanced thermal stability of the enzymatic pattern observed for this clone.

### 3.5. Identification of the Proteins Mapping in the Ox-L and Ox-H Bands

For the identification of guaiacol-oxidizing enzymes mapping in the gel bands Ox-L (see lane C in Figure 6) and Ox-H (see lane B in Figure 6), the corresponding polyacrylamide bands were excised and then subjected to shotgun proteomics. The high-resolution tandem mass spectra corresponding to trypsin-generated peptides were first matched with the hypothetical ORFs from the reference assembled genome of strain *C. trogii* C001 (GCA_020543525.1), as no specific genome annotation was available in public databases for *C. trogii* MUT3379. The genome sequence spanning around the loci encoding the identified peptides was retrieved from the *C. trogii* C001 genome sequence and was then analyzed with AUGUSTUS (http://augustus.gobics.de) for identifying and assembling the exons into potential single mature polypeptides. Matching the identified ORFs was then verified with all the peptides identified by nano LC-MS/MS analysis. Two distinct sets of highly similar MS-certified peptides identified two physically contiguous and closely related genes, encoding hypothetical proteins matching the GMC oxidoreductase, Sequence ID: OSD05574.1 of *Trametes coccinea* BRFM310 (Supplementary Figure S3). The two putative proteins were identified as glucose–methanol–choline (GMC) oxidases, GMC-L and GMC-H. The GMC-L protein (encompassing the genome sequence from 132,412 to 129,808 bp) was identified by peptides deriving from both the Ox-L and Ox-H protein bands. As the size of Ox-L and Ox-H was much different, this convergent identification could indicate alternative splicing, protein aggregation resulting in different migration patterns, or inability to obtain the correct splicing pattern in silico for the GMC oxidase identified in Ox-H. GMC-H, encompassing the genome region from 135,196 to 132,752 bp, was instead only found in the Ox-H protein band. Peptides matching the GMC-L and GMC-H are reported in Figure S4.

Genome mining of the contiguous sequences revealed two other potential GMC oxidases clustered with GMC-L and GMC-H, which we named GMC-EU1 (encompassing the genome sequence from 138,347 to 135,195, with no clear separation from GMC-H start) and GMC-EU2 (encompassing the genome sequence from 126,695 to 124,281 bp) (EU: expression unknown). Only one MS peptide (VVDASVMPLQISAHLSSTLYGIAEK, which was identical to the peptide matching with GMC-H) matched with the GMC-EU1 putative sequence, indicating that these oxidases were probably not significantly produced in our experimental conditions, since specific, unique peptides were not detected. The putative GMC-EU1 best match was with GMC oxidoreductase of *Trametes coccinea* BRFM310 (Sequence ID: OSD05574.1; Identities: 416/633 (66%), Positives: 492/633 (77%), Gaps29/633 (4%)), and the putative GMC-EU2 best match was with GMC oxidoreductase of *Polyporus arcularius* HHB13444 (Sequence ID: TFK84176.1; Identities: 513/625 (82%), Positives: 548/625 (87%), Gaps: 22/625 (3%)). The oxidoreductases described above were all assigned to the alcohol oxidases of the AA3 CAZy subfamily.

In addition to the GMC oxidases described above, a set of other MS peptides, matched with enzymes potentially involved in the metabolism of sugars, is reported in Table S1. It is reasonable to conclude that these enzymes were mapping within the same gel slice, but they were not related to the oxidizing activity observed on guaiacol as a substrate.

## 4. Discussion

Microbial division of labour has been described in different types of microorganisms, from unicellular to multicellular ones [4,26]. In filamentous microorganisms, this phenomenon allows cell populations within the mycelium to compartmentalize explorative, assimilative, and sporulating functions, thus favouring their evolutionary adaptation to environmental changes. Cellular cytoplasmic continuity within their hyphae further allows the building of a complex mycelium net that can continuously be remodelled thanks to an optimal distribution of metabolic resources [27,28]. By assuming that the fungal mycelium of *C. trogii* MUT3379 could contain genetically different nuclei (which may result from spontaneous mutations or acquisition through hyphal fusion and heterokaryosis [13,29]), herein we attempted to separate distinct cell populations by protoplast formation and regeneration.

Initial analysis revealed distinct morphological differences among the protoplast-derived clones during submerged fermentations, with some clones showing a so-called dispersed mycelium, while others presented rounded packed mycelial pellets [30]. These morphological differences have been traditionally attributed to the culturing methods used [31]. However, recent studies demonstrated that genetically modified mutants are programmed to differentiate diverse morphological structures [32]. In our case, we can speculate that the observed morphological differences might be attributed to variations in the chemical-physical characteristics of the cell walls of the clones, as previously observed in *Aspergillus* species [33]. In our protoplast-derived clones, phenotypical differences were also observed in cultivation broths’ pigmentation, linked to the differential production of coloured metabolites, such as melanin-like pigments. Notably, a correlation between pellet morphology and pigmentation was observed: for example, clones producing dark pigments presented larger, heterogeneously shaped pellets. The synthesis of different molecules (eventually also pigments) could determine alterations of charges and consequently guide the formation of one mycelial structure rather than another [34,35,36]. In addition, more compacted pellets could act as a physical barrier which limits the uptake of nutrients and oxygen, and the resulting nutritional stress could trigger various metabolic responses, including melanin production. Under these stress conditions, pigment production might be more likely than in other isolated clones [34,35,36].

Differences in ABTS oxidizing activity were also observed, from clones with low oxidative potential to high-producing ones. Two clones, 91p and 100p, were found to produce 45% and 34%, respectively, more ABTS oxidizing activity than the native strain MUT3379. Typically, metabolite/enzyme production titer in microbial cultures is assessed as the average output of the entire culture. Thus, the presence of cells with low production capacities can significantly reduce the overall production [37]. Through protoplast preparation, the removal of these clones could ideally be possible, leading to the selection of lineages with high-production capacities, as in the case of 91p and 100p clones. In a similar way, Herpoël et al. (2000) explored this concept in *Pycnoporus cinnabarinus*: the authors were able to improve the laccase production of the parental strain (equal to 11,000 U/L) by isolating a high-producing monokaryotic clone, with productivities as high as 29,000 U/L [38]. However, our use of protoplasts, instead of spores as a method of isolating clonal populations, came from the idea that sporulating hyphae specialize in producing spores as a mechanism of division of labour [39]. Vegetative hyphae, which indeed specialize in metabolically supporting the sporulating population, potentially harbour more interesting nuclei for industrial production. Although the genetic determinants of the high-producing features of clones 91p and 100p are still to be elucidated, we can speculate that they could harbour mutations or gene duplication that favour enzyme production [40]. In addition, even if in filamentous fungi division of labour is traditionally attributed to differential gene expression [41,42], evidence also suggests that genomic instability could play a role, as previously observed for filamentous actinomycetes [28]. Indeed, in the mycorrhizal fungus *Glomus irregulare,* a high genetic variability was observed from the germination of single spores, resulting in phenotypically distinct variants of the fungus [43]. This genetic variation within the mycelium might influence the ability of the fungus to adapt to environmental changes as well as to influence its symbiotic relationships [44]. Another hypothesis is related to the mycelium morphology observed for 91p and 100p clones, both belonging to the group featured by a high abundance of small mycelium clumps in the fermentation broth. Given that enzyme secretion is believed to occur at the tips of the growing hyphae [45,46], the higher abundance of mycelium clumps with dispersed hyphae in these liquid cultures might contribute to their higher production levels.

Besides the differential productivity of ABTS oxidase activity among different protoplast-derived clones, in a limited number of isolates, a qualitative difference in the produced enzymatic pattern was also observed. Fungal laccases are produced by multiple genes within the strain’s genome [47]. These genes often give rise to different forms of laccase enzymes, which are typically expressed as two or more distinct isoenzymes [48]. Each isoenzyme may vary in its structure or function, allowing the fungus to adapt to different environmental conditions or to perform diverse physiological roles throughout the fungal life cycle [47,49]. For instance, genomic analysis of the *Pleurotus eryngii* laccase gene family has identified at least 10 laccase isoenzymes. Among these, three are likely closely associated with lignocellulose degradation, while others play essential roles in growth and development [49]. Sequence analysis and heterologous expression of *Cerrena* sp. HYB07 laccase gene family revealed isoenzymes with variable substrate-binding loops and differing optimal temperatures, suggesting diverse substrate ranges and catalytic properties [50]. Different forms of oxidative enzymes could also be found in the lignin-degrading peroxidases gene family of *Trametes hirsuta* 072 [51]. Even for this class of oxidative enzymes, up to 18 genes encoding peroxidases were found within the strain genome. Interestingly, the expression of these genes significantly varied under different culturing conditions, and the secretome analysis revealed that only a subset was secreted, often in multiple isoforms.

Through protoplast manipulation, we succeeded in separating clones of *C. trogii* with the capacity of producing different guaiacol-oxidative patterns. Thanks to the approach used, we have evidenced a cluster of at least four closely related GMC-oxidases, which were differentially expressed, and which were never observed in our previous studies. We can speculate that through protoplast preparation, the removal of the dominant “oxidative enzymes profiles” was possible and gave a relatively homogeneous culture producing to date “hindered” oxidative enzymes. Although further analyses are required to shed light on the genetic basis of the observed phenotypic variations, the different features as pH range and temperature tolerance, observed in the oxidase activities of the secretomes in clone 6p and clones 26p (in which the different GMC-oxidase partners were observed) supported our initial hypothesis of a division of labour within the mycelium. Besides the ecological meaning of our result, this method may be useful for identifying and producing novel isoenzymes endowed with unique properties for potential industrial applications, paving the way for a new approach to enzyme mining.

## 5. Conclusions

In the production of enzymes, native fungal strains are sometimes preferred to recombinant strains because they can achieve high yield productions while being cultivated on cheap substrates, reducing costs. The drawback is that fungi have an intrinsic variability which can pose serious limits to reproducibility in industrial productions. In this work, we investigated the possibility of isolating clonal populations of *C. trogii* MUT3379 by protoplast preparation and regeneration. This approach allowed us to (i) considerably improve the production of the ABTS oxidase activity in the Lac3379-1-producing strain, and (ii) to highlight the presence of novel oxidative enzyme variants not significantly produced in the native strains or that are being hindered by major isoforms. From an industrial perspective, the possibility of selecting, preserving, and fermenting these different lineages offers a great advantage in maximizing strain improvement and maintenance processes, along with the advantage of isolating clones able to produce qualitatively different enzymes. The approach used in this work could be easily extended to other families of enzymes and to other metabolites of fungal origin.

## Data Availability

The original contributions presented in this study are included in the article/supplementary material. Further inquiries can be directed to the corresponding author.

## Supplementary Materials

The following supporting information can be downloaded at: https://www.mdpi.com/article/10.3390/jof11120890/s1, Figure S1: *C. trogii* MUT3379 (A) and three selected protoplast-derived clones (B–D) grown on YMG plates, exemplifying the morphological differences upon growth on agar solid medium. Figure S2: Details of three representative protoplasts-regenerated clones cultivated in liquid cultures: examples of variable broth pigmentations (B–D) and different mycelium pellets’ morphology (E,F), in comparison to the control strain (A). Figure S3: CLUSTAL 2.1 multiple sequence alignment of GMC-L, GMC-H, and GMC-BRFM310 (GMC oxidoreductase Sequence ID: OSD05574.1 of Trametes coccinea BRFM310). GMC-L vs. GMC-BRFM310: Identities 430/631 (68%), Positives 500/631 (79%), Gaps 36/631 (5%); GMC-H vs. GMC-BRFM310: Identities 447/591 (76%), Positives 517/591 (87%), Gaps 8/591 (1%). Figure S4: LC-MS/MS identified peptides matching with GMC-L putative oxidase (A) and with GMC-H putative oxidase (B). Differences in LC-MS/MS identified peptides, suggesting that GMC-L and GMC-H are coproduced, are marked in red. Table S1: Summary of enzymes potentially involved in the metabolism of sugars, which were identified by the LC-MS/MS analysis of the gel bands Ox-H and Ox-L.
