# Supplementary material for: Unravelling the Potential of Fungal Division of Labour in the Laccase Producer Coriolopsis trogii MUT3379 Through Protoplast Formation and Regeneration
Source: J Fungi (Basel). 2025 Dec 17;11(12):890. doi: 10.3390/jof11120890 (PMC12733382; doi:10.3390/jof11120890)
Supplement: Supplementary file 1 [file jof-11-00890-s001.zip › jof-4004146-supplementary.pdf]

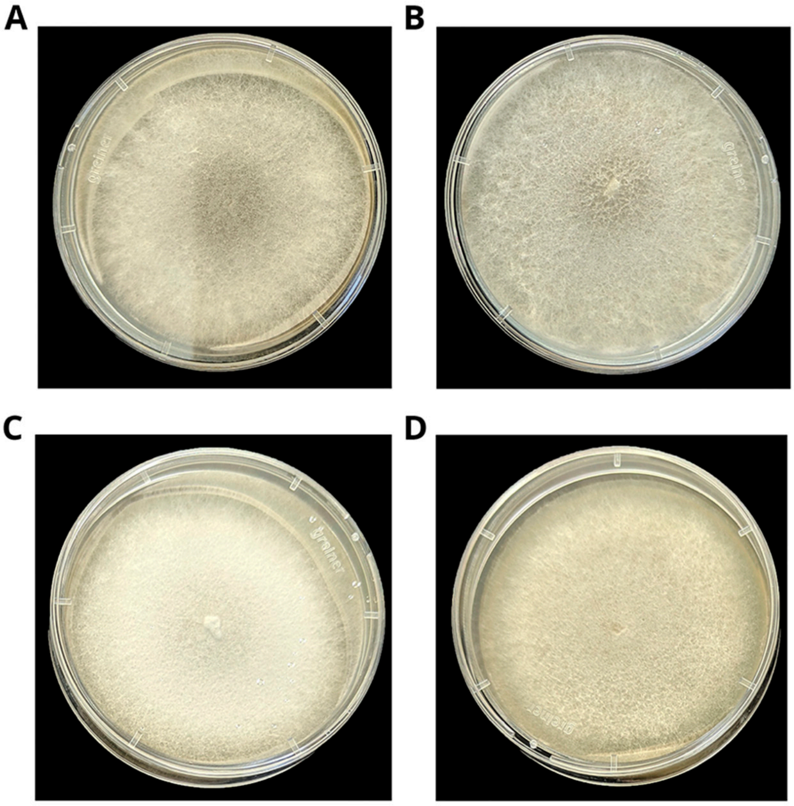

Figure S1. *C. trogii* MUT3379 (A) and three selected protoplasts-derived clones (B-C-D) grown on YMG plates, exemplifying the morphological differences upon growth on agar solid medium.

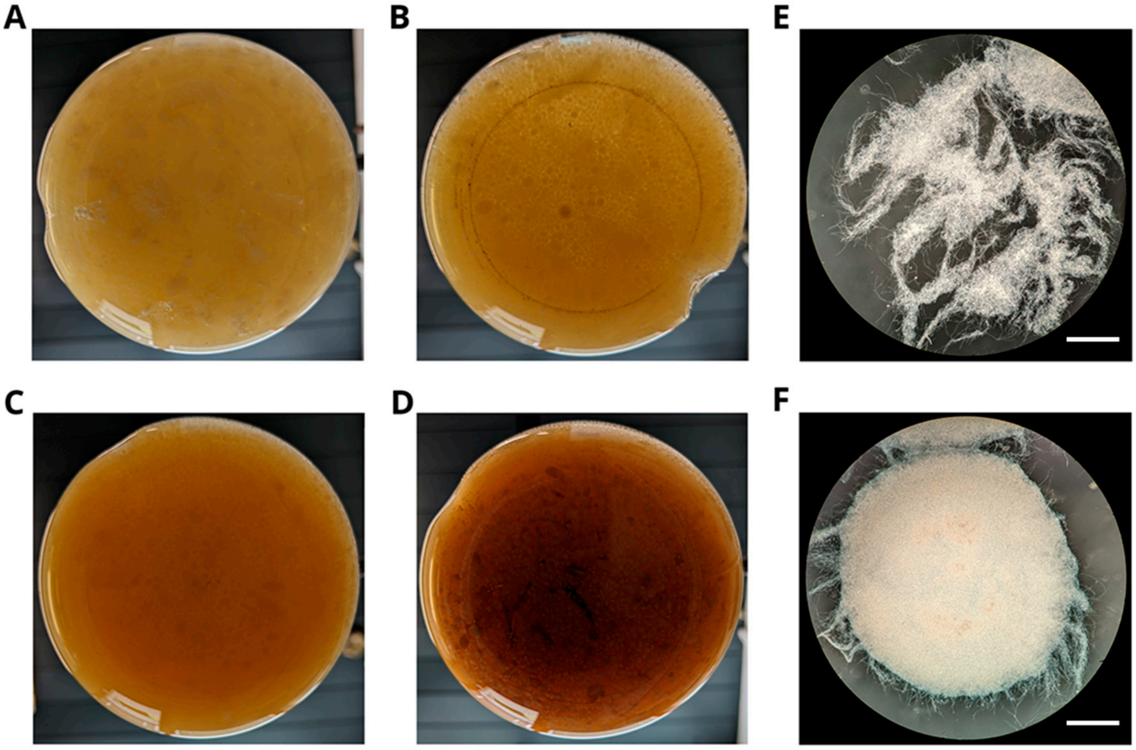

Figure S2. Details of three representative protoplasts-regenerated clones cultivated in liquid cultures: examples of variable broth pigmentations (B, C, D) and different mycelium pellets' morphology (E, F), in comparison to

the control strain (A). Panels a-to-d show views from the bottom of the flasks of submerged cultures, whereas in panels e and f picture taken at optical microscope are reported (size bar 100 μm).

```
GMC-L.            ----------------MYISVRQYAKAIPCSSGITSNATSVASQTYDYIIVGGGLTGVTV
GMC-H.            MLSRRFRSLLTTLPVVAISVGFQCTEGVTIPSGITTDTTAIANQTFDYIIVGGGLTGVTV
GMC-BRFM310.      MLRLPLPVRIGVIALLARCALAIPQVHNRASSGITSDPSAVDGQTYDYIVVGGGLTGTTV
                                                .****::.::: .**:***:*******.**

GMC-L.            AARLAEDPALKILLIEAGGDNRTNPQVYDIQQFTVAFNGPLDWAWTSDYGKIIHGGKTLG
GMC-H.            AARLAEDSSLKILLIEAGGDDRANSQVYDIYQYSAAFNGPLDWTWVSDRGKIIHGGKTLG
GMC-BRFM310.      AARLAENSALKILLIEAGGDDRTNPQIYDIYEYSQVFNGPLDWAWEADQGKIIHGGKTLG
                  ******:.:***********:*:*.*:*** ::: .*******:* :* ***********

GMC-L.            GSSSINGAAWTRGQKAQYDAWSTLLEESEASVGWNWHGLFTYMKQAETFTPPNAQQRAKG
GMC-H.            GGSSINGGAWTRGLKAQYDSWSALLEPSEVSVGWNWDGVFEYMKKAETFSPPNAQQQAKG
GMC-BRFM310.      GSSSINGAAWTRGLNAQYDSWSSLLEPEEASVGWNWDNLFTYMKKAEAFSAPNDQQRAKG
                  *.*****.***** :****:**:*** .*.******..:* ***:**:*:.** **:***

GMC-L.            ADYNSSYHGNAGPVQAAFPADIFGGPPMPAFVNSVVNVTGIDLSRDLNGGMPNCVSYTPL
GMC-H.            ADSIASYHGTSGPVQATFPDLMYGGPQMKDFVDTVVNVTGIKHFKDLNGGTPNCVSYTPL
GMC-BRFM310.      ADSIASYHGTAGPVQATFPDAMYGGPQMPAFVNTVVNVTGMPHYRDLNGGTPNCVSITPL
                  **  :****.:*****:**  ::*** *  **::******:   :***** ***** ***

GMC-L.            SLNPHEEDRRSSSIEAYYTPIEASTKGLTLLIEHQVTRILFTDSSNVPHFATGVEFAASD
GMC-H.            SLNWHKGDNRSSSIEAYYTPVEGRRKGWTLLVKHQVTRILFANSSSLPHVATGVEFAHAD
GMC-BRFM310.      SINWHADDHRSSSIEAYYTPVENSRQGWTLLIQHMATKILFDG-TSAPLTAVGVEFGASD
                  *:* *  *.***********:*   :* ***::* .*:*** . :. *  *.****. :*

GMC-L.            GSGPRLKVYARKEIILAAGAIQTPALLQHSGIGDSAILGPLGINTLVDLKTVGRNFQEQT
GMC-H.            GSGARVKAFARKEIIIAAGAIQTPALLQLSGIGDSAVLEPLGISTLIDLKTVGRNFQEQT
GMC-BRFM310.      GTGSRYKAFARKEVILAAGAIQTPALLQLSGIGDSAILGPLGINTLIDLKTVGKNLQEQT
                  *:*.* *.:****:*:************ *******:* ****.**:******:*:****

GMC-L.            QSLIGAMGNGFEVGGRGPTDVIAFPNIRQVFGNQANASIQKIQQSLSAWAESQSIN-GHS
GMC-H.            QSALGADGNGFNPGGRGPTDAIAFPNIRQVFGNKANASIQKIQQSLSTWAESQSNN-GLS
GMC-BRFM310.      QNSIGAKGNGFNPGGRGPTDAIAFPNIYQVFGSQAASAVRTIQSSLSSWAQSQAASGALS
                  *. :** ****: *******.****** ****.:* ::::.**.***:**:**: . . *

GMC-L.            KEALEEIFRIQADLIVKKD--APVVELFFLTAPDPEVIDIAMWPLLPFSRGNVTITSSDA
GMC-H.            KGALEEIFRIQADLIINEDGVAPVVELFFDSG-YPDDIGIVMWPLLPFSRGNVTVKSKDP
GMC-BRFM310.      ADALNTIFGIQADLIINKN--APVVELFFDSG-FPDDIGIVMWPLLPFSRGNVTIQSNNP
                    **: ** ******::::  ******** :.  *: *.*.*************: *.:.

GMC-L.            FVKPNVIVNYFSVDWDLDVHVAGARLSRLVLDNPPLS---ILPPISHYLPGPLAFFALTS
GMC-H.            FAKPVVTVNYFSVDWDLDVHVAGSRLSRIILASPPLS---NLR-----------------
GMC-BRFM310.      FAKPSVNVNYFSVDFDLTMHIAGARLSRKLLASPPLSTLS--------------------
                  *.** * *******:** :*:**:**** :* .****

GMC-L.            YHTHPSALRSSAGELVPGLATVPDDGHGGTDEDWKAWILQAGDGVGFDAVSHPIGTAAMM
GMC-H.            ---------SAKGETVPGLATVPDDGHGGTDTDWKNWILNP--SAGFGAVSHPIGTAAMM
GMC-BRFM310.      -----------LGETVPGFQTVPDNGNGGSDADWKKWILKPGNSAGFASVAHPIGTAAMM
                              ** ***: ****:*:**:* *** ***:.  ..** :*:*********

GMC-L.            RRSLGGVVDAHLRVYDTANVRVVDASVMPMQVSAHLSSTLYGVAEKAADLIKADRALGR
GMC-H.            RRSLGGVVDAQLRVYDTANVRVVDASVMPLQISAHLSSTLYGIAEKAADLIKQGH----
GMC-BRFM310.      KRSLGGVVDAQLKVYDTTNLRVVDASMMPLQISAHLSSTLYGVAEKAADLIKAAQ----
                  :*********:*:****:*:******:**:*:**********:*********  :
```

Figure S3. CLUSTAL 2.1 multiple sequence alignment of GMC-L, GMC-H, and GMC-BRFM310 (GMC oxidoreductase Sequence ID: OSD05574.1 of *Trametes coccinea* BRFM310). GMC-L vs GMC-BRFM310: Identities 430/631(68%), Positives 500/631(79%), Gaps 36/631(5%); GMC-H vs GMC-BRFM310: Identities 447/591(76%), Positives 517/591(87%), Gaps 8/591(1%).

A

```
  1-MYISVRQYAKAIPCSSGITSNATSVASQTYDYIIVGGGLTGVTVAARLAEDPALKILLIEAGGDNRTNPQVYDIQQFTVAFNGPLDWAWTSDYGKIIHGG
___________________________________________________LAEDPALK_____________________________________________
___________________________________________________________ILLIEAGGDNR__________________________________

101-KTLGGSSSINGAAWTRGQKAQYDAWSTLLEESEASVGWNWHGLFTYMKQAETFTPPNAQQRAKGADYNSSYHGNAGPVQAAFPADIFGGPPMPAFVNSVV
_____TLGGSSSINGAAWTR____________________________________________________________________________________

201-NVTGIDLSRDLNGGMPNCVSYTPLSLNPHEEDRRSSSIEAYYTPIEASTKGLTLLIEHQVTRILFTDSSNVPHFATGVEFAASDGSGPRLKVYARKEIIL

301-AAGAIQTPALLQHSGIGDSAILGPLGINTLVDLKTVGRNFQEQTQSLIGAMGNGFEVGGRGPTDVIAFPNIRQVFGNQANASIQKIQQSLSAWAESQSIN
________________________________________________________________GPTDVIAFPNIR_____________IQQSLSAWAESQSIN

401-GHSKEALEEIFRIQADLIVKKDAPVVELFFLTAPDPEVIDIAMWPLLPFSRGNVTITSSDAFVKPNVIVNYFSVDWDLDVHVAGARLSRLVLDNPPLSIL
________EALEEIFR________________________________________________________________________________________
________________IQADLIVKK_______________________________________________________________________________
____GHSK________IQADLIVK _______________________________________________________________________________
________________

501-PPISHYLPGPLAFFALTSYHTHPSALRSSAGELVPGLATVPDDGHGGTDEDWKAWILQAGDGVGFDAVSHPIGTAAMMRRSLGGVVDAHLRVYDTANVRV
___________________________________________________________________________________RSLGGVVDAHLR________V
____________________________________________________________________________________SLGGVVDAHLR_________
_______________________________________________________________________________________________VYDTANVR_

601-VDASVMPMQVSAHLSSTLYGVAEKAADLIKADRALGR
____VDASVMPMQVSAHLSSTLYGVAEK_____________
____________________________AADLIKADR____
```

B

```
  1-MLSRRFRSLLTTLPVVAISVGFQCTEGVTIPSGITTDTTAIANQTFDYIIVGGGLTGVTVAARLAEDSSLKILLIEAGGDDRANSQVYDIYQYSAAFNGP
___________________________________________________________________LAEDSSLK_____________________________
___________________________________________________________________________ILLIEAGGDDR__________________

101-LDWTWVSDRGKIIHGGKTLGGGSSINGGAWTRGLKAQYDSWSALLEPSEVSVGWNWDGVFEYMKKAETFSPPNAQQQAKGADSIASYHGTSGPVQATFPD
_____________________TLGGGSSINGGAWTR____________________________________________________________________

201-LMYGGPQMKDFVDTVVNVTGIKHFKDLNGGTPNCVSYTPLSLNWHKGDNRSSSIEAYYTPVEGRRKGWTLLVKHQVTRILFANSSSLPHVATGVEFAHAD

301-GSGARVKAFARKEIIIAAGAIQTPALLQLSGIGDSAVLEPLGISTLIDLKTVGRNFQEQTQSALGADGNGFNPGGRGPTDAIAFPNIRQVFGNKANASIQ
__________________________________________________________NFQEQTQSALGADGNGFNPGGR________________________

401-KIQQSLSTWAESQSNNGLSKGALEEIFRIQADLIINEDGVAPVVELFFDSGYPDDIGIVMWPLLPFSRGNVTVKSKDPFAKPVVTVNYFSVDWDLDVHVA
_____IQQSLSTWAESQSNNGLSK________________________________________________________________________________
________________________GALEEIFR________________________________________________________________________

501-GSRLSRIILASPPLSNLRSAKGETVPGLATVPDDGHGGTDTDWKNWILNPSAGFGAVSHPIGTAAMMRRSLGGVVDAQLRVYDTANVRVVDASVMPLQIS
_________________________________________________________________________SLGGVVDAQLR________VVDASVMPLQIS
________________________________________________________________________RSLGGVVDAQLR____________________
____________________________________________________________________________________VYDTANVR____________

601-AHLSSTLYGIAEKAADLIKQGH
____AHLSSTLYGIAEK_________
_________________AADLIKQGH
```

Figure S4. LC-MS/MS identified peptides matching with GMC-L putative oxidase (A) and with GMC-H putative oxidase (B). Differences in LC-MS/MS identified peptides suggesting that GMC-L and GMC-H are co-produced are marked in red.

Table S1. Summary of enzymes potentially involved in the metabolism of sugars which were identified by the LC-MS/MS analysis of the gel bands Ox-H and Ox-L.

Guaicol positive band	Region of interest in the *C. trogii* C001 (GCA_020543525.1) genome (#start-#stop)	Best match in databases	Number of LC-MS matching peptides	Ratio between identified amino acids and total amino acids in the homologous protein

Ox-H	1104522-1102283	bacterial alpha-L-rhamnosidase domain protein [*Fomes fomentarius*] Sequence ID: KAI0762626.1	4	64/671
Ox-H	1031652-1029841	glycoside hydrolase [*Daedaleopsis nitida*] Sequence ID: KAI0757206.1	7	102/435
Ox-H	3531971-3533523	Cellobiohydrolase I [*Fomes fomentarius*] Sequence ID: KAI0800646.1	13	178/456
Ox-H	3575749-3574014	glycoside hydrolase family 5 protein [*Polyporus arcularius* HHB13444] Sequence ID: TFK84199.1	4	56/424
Ox-L	2263531-2261066	chondroitin AC/alginate lyase [*Ganoderma leucocontextum*] Sequence ID: KAI1789808.1	3	33/762
Ox-L	1263847-1261789	Glucanosyltransferase-domain-containing protein [*Ganoderma leucocontextum*] Sequence ID: KAI1797587.1	4	52/555
Ox-L	3531971-3533523	Cellobiohydrolase I [*Fomes fomentarius*] Sequence ID: KAI0800646.1	3	55/456
Ox-L	2478256-2475919	glycoside hydrolase family 16 protein [*Cerioporus squamosus*] Sequence ID: KAI0698410.1	2	35/599
